# Cudraxanthone D Regulates Epithelial-Mesenchymal Transition by Autophagy Inhibition in Oral Squamous Cell Carcinoma Cell Lines

**DOI:** 10.1155/2019/5213028

**Published:** 2019-10-31

**Authors:** Su-Bin Yu, Hae-Mi Kang, Dan-Bi Park, Bong-Soo Park, In-Ryoung Kim

**Affiliations:** ^1^Department of Oral Anatomy, School of Dentistry, Pusan National University, 49 Busandaehak-ro, Mulguem-eup, Yangsan-si, Gyeongsangnam-do 50612, Republic of Korea; ^2^BK21 PLUS Project, School of Dentistry, Pusan National University, 49 Busandaehak-ro, Mulguem-eup, Yangsan-si, Gyeongsangnam-do 50612, Republic of Korea; ^3^Dental and Life Science Institute, Pusan National University, 49 Busandaehak-ro, Mulguem-eup, Yangsan-si, Gyeongsangnam-do 50612, Republic of Korea

## Abstract

Cudraxanthone D (CD), derived from the root bark of *Cudrania tricuspidata*, is a natural xanthone compound. However, the biological activity of CD in terms of human metabolism has been barely reported to date. Autophagy is known as a self-degradation process related to cancer cell viability and metastasis. Herein, we investigated the effects of CD on human oral squamous cell carcinoma (OSCC) metastatic related cell phenotype. We confirmed that CD effectively decreased proliferation and viability in a time- and dose-dependent manner in human OSCC cells. In addition, OSCC cell migration, invasion, and EMT were inhibited by CD. To further determine the underlying mechanism of CD's inhibition of cell metastatic potential, we established the relationship between EMT and autophagy in OSCC cells. Thus, our findings indicated that CD inhibited the potential metastatic abilities of OSCC cells by attenuating autophagy.

## 1. Introduction

Autophagy is a biological process that protects against cellular homeostasis disruption from intracellular or environmental stresses, such as hypoxia, starvation, and cytochemism [1]. During autophagic processes, double-membrane vesicles, called autophagosomes, sequester cytoplasmic organelles and carry them to lysosomes. Lastly, autophagosome-fused lysosomes are degraded [2]. Autophagy is related to many pathological and physiological processes, such as apoptosis and cancer metastasis. The role of autophagy in tumor cells is known as a “dual-edged sword”—it may function as a tumor suppressor by protecting proteins from damage during the tumor formation stage, while in terms of tumor growth, autophagy might be a tumor promoter by distributing substrates for metabolic balance. Recently, much evidence of autophagy function in cancer has been reported such that it is key in the development of chemotherapeutic agents [3–6].

Tumor metastasis is divided into sequences of stages. Of the multistep processes, epithelial-mesenchymal transition (EMT) is concerned with local invasion [7], by which, when it is initiated, epithelial cells lose their properties and are transformed into different types of cells with mesenchymal properties, thereby increasing cell mobility and migration [8]. The interplay between autophagy and EMT has been thought for a long time to be connected. One study reported that increasing autophagy supports various cancer cells' EMT during spreading by upregulating cell motility [9–11]. On the other hand, there are data that indicate that autophagy prevents tumor cells from engaging in EMT by activating autophagic process and returning to the EMT phenotype. Synthetically, it is important to control autophagy and EMT interactions for regulation of tumor metastasis [12–14].

Cudraxanthone D (CD), a natural xanthone compound, is derived from the root bark of *Cudrania tricuspidata* distributed widely in Korea, Japan, and China [15]. Its compounds are used as traditional drugs for many diseases [16–19]. However, the relationship among oral squamous cell carcinoma (OSCC), autophagy, and EMT is unknown. So, in this study, we investigate what the role of autophagy is in OSCC cell metastatic cell phenotype and elucidate whether CD might be a strategically novel agent in oral cancer cell migration and invasion.

As is well known, autophagy and EMT are major biological processes in the occurrence and development of cancer, and there is a complex relationship between autophagy and EMT signaling pathways.

## 2. Materials and Methods

### 2.1. Reagents

Fetal bovine serum (FBS), Dulbecco's modified Eagle medium (DMEM), and DMEM : nutrient mixture F-12 (DMEM/F-12) were obtained from Thermo Fisher Scientific (Pittsburgh, USA). CD was purchased from ChemFace (Wuhan, China). 3-[4,5-Dimethylthiazol-2-yl]2,5-diphenyl tetrazolium bromide (MTT) and dimethyl sulfoxide (DMSO) were procured from Duchefa Biochemie (Haarlem, the Netherlands). Rabbit antibodies, E-cadherin, slug, snail, ATG5, beclin-1, and LC3B were purchased from Cell Signaling Technology (Beverly, USA). The anti-mouse HRP-conjugated secondary antibody and anti-rabbit HRP-conjugated secondary antibody were obtained from Enzo Biochem (Farmingdale, USA). Acridine orange and 3MA (3-methyladenine) were obtained from Sigma-Aldrich (St. Louis, USA).

### 2.2. Cell Culture

The Ca9-22, SCC25, and CAL27 cells were procured from America Type Culture Collection (Manassas, USA). HSC4 cells were provided by Professor Sung-Dae Cho of the Department of Oral Pathology, School of Dentistry, Chonbuk National University (Jeonju, Korea). Ca9-22 and CAL27 cells were cultured in DDMEM, Thermo Fisher Scientific (Pittsburgh, USA), while the SCC25 cells were cultured in DMEM/F-12. HSC4 cells were cultured in MEM with 10% FBS and 1% penicillin-streptomycin at 37°C in a humidified 5% carbon dioxide (CO_2_) atmosphere. CD was dissolved in DMSO at a stock solution of 10 mM and was kept at 4°C. The CD stock solution was diluted to marked concentrations with medium when we required.

### 2.3. Cell Viability Measurement

The cell viability of OSCC cells was determined using an MTT assay. Cells were cultured in 96-well plates and then incubated for different time periods in the presence of various concentrations of CD (0–100 *μ*M). After terminating the treatment, the medium was removed and 100 *μ*l of MTT (500 mg/mL) was added to each well. The cells were incubated for 3 h at 37°C to form formazan crystals. The formazan crystals were measured as described in the previous study [20].

### 2.4. Wound Healing Assay

A wound healing assay was performed to investigate the cell proliferation and migration ability of OSCC cells. Cells were seeded to roughly 80–90% confluence into six-well plates. A 1 mm pipette tip was used to formulate wounds, and debris was washed with PBS twice. Migrated cells into the wound area were visualized under an inverted microscope at 200x magnification (Olympus, Tokyo, Japan).

### 2.5. Migration and Invasion Assay

Cell invasion assay (transwell assay) and migration assay were conducted to examine the capacity of cell invasion and migration, as described previously. A transwell with an 8 *μ*m pore polycarbonate membrane (Corning Costar, Cambridge, USA) was coated with 40 *μ*l Matrigel at 200 *μ*g/ml and incubated for 2 h. Next, OSCC cells were seeded and treated with 3MA, resveratrol, and CD. The upper chamber of the transwell was filled with serum-free medium and the lower chamber with 800 *μ*l medium containing 10% FBS for 72 h of incubation at 37°C in a humidified 5% CO_2_ atmosphere. The cells were fixed in methanol and stained with hematoxylin for 30 min and were then counted under an inverted microscope (Olympus, Tokyo, Japan).

### 2.6. Immunofluorescent Staining

OSCC cells were cultured in DMEM with 3MA, resveratrol, and CD on a Lab-Tek™ II Chamber Slide (Nunc; Thermo Fisher Scientific, Rochester, USA). After 24 h, cells were stained with acridine orange (Sigma-Aldrich, St. Louis, USA). Fluorescent images were observed and analyzed as described in the previous study [20].

### 2.7. Western Blot Assay

Cells were harvested in the form of pellets, and then it was lysed in 150 *μ*l RIPA buffer (300 mM NaCl, 50 mM Tris-Cl (pH 7.6), 0.5% Triton X-100, 2 mM PMSF, 2 *μ*l/ml aprotinin, and 2 *μ*l/ml leupeptin/protease inhibitor cocktail) and incubated at 4°C for 1 h. The lysate was centrifuged at 13,200 RPM for 30 minutes at 4°C. Protein quantification, electrophoresis, and detection of protein expression were performed in the same manner as described in the previous study [20].

### 2.8. Statistical Analysis

All data represent mean ± SD (standard deviation). Statistical analyses were conducted using one-way analysis of variance (ANOVA) followed by Dunnett's multiple comparison test. A difference of *p* value less than 0.05 was considered significant.

## 3. Results

### 3.1. Cudraxanthone D Affects Cell Viability in Human OSCC Cell Lines

**Figure 1:**
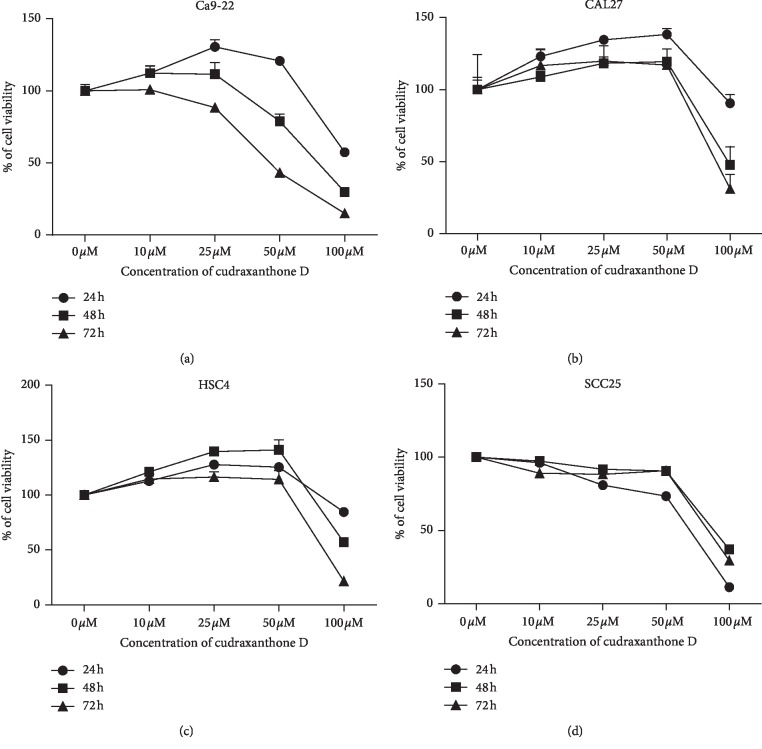
The effect of CD on cell viability in OSCC cells. OSCC cells were treated with CD (0–100 *μ*M) for 24–72 h. (a) Ca9-22, (b) CAL27, (c) HSC4, and (d) SCC25. Each experiment was performed in triplicate. Error bars denote mean ± SD (standard deviation). ^*∗*^*p* < 0.05 versus untreated cell, respectively.

To choose proper concentrations of CD, human OSCC cell lines, specifically Ca9-22, CAL27, SCC25, and HSC4 cells, were cultured with 0–100 *μ*M CD for 24–72 h. After treatment, cell viability was determined using an MTT assay. As shown in Figure 1, CD was cytotoxic to OSCC cells in a dose- and time-dependent manner. In particular, Ca9-22 and SCC25 cells were very sensitive to CD. So, these cells were selected and the subsequent experiments were carried out.

### 3.2. Cudraxanthone D Suppresses Epithelial-Mesenchymal Transition in Ca9-22 and SCC25 Cells

**Figure 2:**
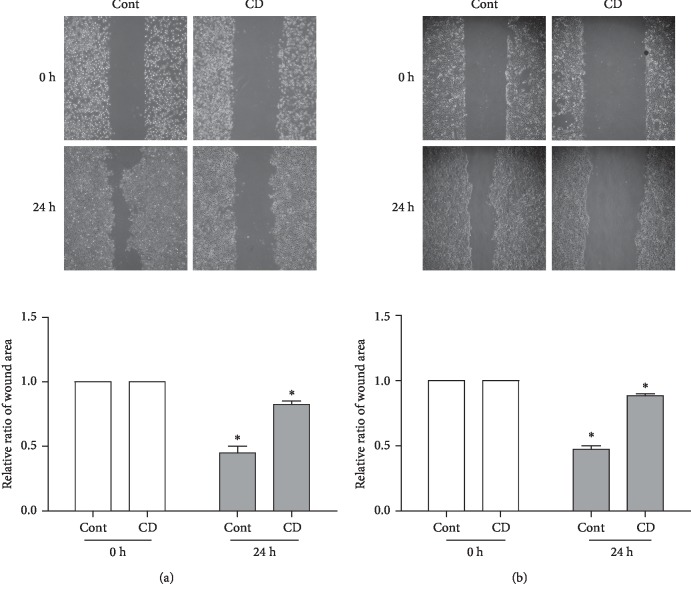
CD suppressed wound healing in OSCC cell lines. (a) Representative images and graphs of Ca9-22 cells' wound healing area treated with 50 *μ*M CD for 24 h. (b) Representative images and graphs of SCC25 cells' wound healing area treated with 50 *μ*M CD for 24 h. Error bars represent mean ± standard deviation (SD). ^*∗*^*p* < 0.05 versus 0 h respectively.

**Figure 3:**
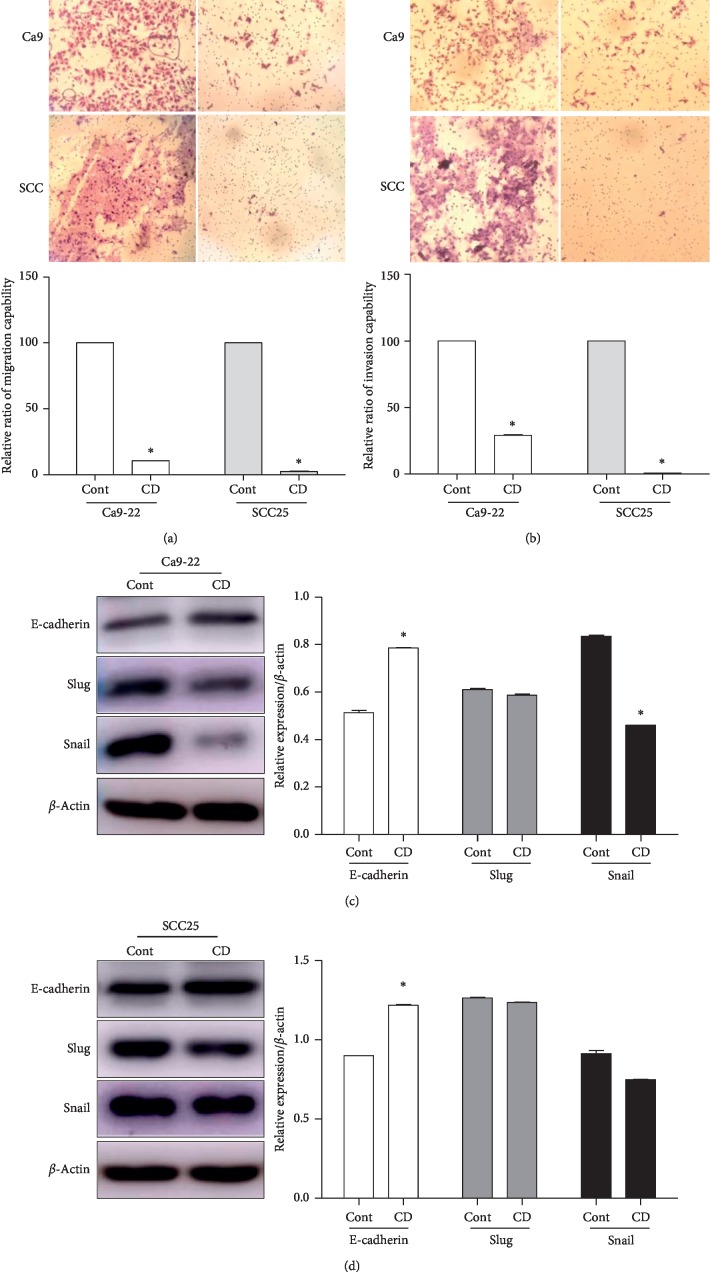
CD inhibited EMT in human OSCC cell lines. Migration assay (a) and invasion assay (b) with Ca9-22 and SCC25 cells were conducted with a transwell chamber in the absence or presence of 50 *μ*M CD for 24 h. Protein expression of EMT markers (E-cadherin, slug, and snail) was investigated after CD treatment in Ca9-22 (c) and SCC25 (d) cells. Relative protein expression levels were normalized to *β*-actin. Error bars represent mean ± standard deviation (SD). ^*∗*^*p* < 0.05 versus control, respectively.

To determine whether CD affected OSCC cell cancer metastatic phenotype, we conducted a wound healing assay, transwell assay, and western blotting. Cell migration capabilities were investigated by the wound healing assay and transwell assay without Matrigel. The results demonstrated that the scratch wound area of the nontreated group was covered with proliferating Ca9-22 and SCC25 cells by approximately 55% while in the CD-treated group, the wound area covered only approximately 10% and 11% compared to prescratching, respectively (Figures 2(a) and 2(b)). In addition, we performed the transwell assay to verify migration capability in the absence of Matrigel, also known as a migration assay. First, Ca9-22 and SCC25 cell suspensions were located in the upper chamber with serum-starved media, while the lower chamber was filled with normal media. After 24 h, the nontreated group of Ca9-22 and SCC25 cells was found on the underside of the transwell filter. However, with the CD-treated group, there was a dramatic reduction in the migration capability of both cells by approximately 10% and 2%, respectively (Figure 3(a)). Similar results were found with the invasion assay, with applied Matrigel in the upper chamber. The CD-treated group exhibited inhibited invasion capabilities for both cells compared to the control groups (Figure 3(b)). Moreover, we confirmed the expression of EMT-associated proteins, such as E-cadherin, slug, and snail as shown in Figures 3(c) and 3(d). CD significantly upregulated E-cadherin, while slug expression was scarcely changed in both cells. Variation in snail expression was observed quite pronouncedly in Ca9-22. Collectively, these results suggested that CD effectively suppressed the EMT of OSCC cell lines.

### 3.3. Suppression of Resveratrol-Induced Autophagy by Cudraxanthone D Affected EMT in Ca9-22

**Figure 4:**
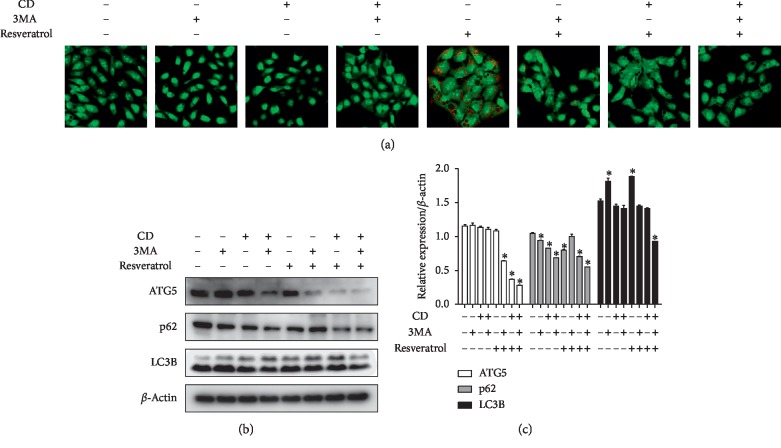
CD acts as an autophagy inhibitor in resveratrol-induced autophagy in Ca9-22 cells. (a) Cells were pretreated with 3MA (1 mM) for 2 h and then 25 *μ*M resveratrol and 50 *μ*M CD for 24 h. After treatment, AO-stained cells were observed under a confocal microscope. (b) Autophagy-related protein expression was confirmed by western blot. Each group was treated at the indicated concentrations and times. (c) Relative protein expression levels were normalized to *β*-actin. Error bars represent mean ± standard deviation (SD). ^*∗*^*p* < 0.05 versus control, respectively.

A variety of evidence indicated that autophagy induction is associated with tumor cell EMT in various cancers [5, 14]. Hence, in order to analyze the underlying mechanisms of CD-inhibited EMT, among diverse autophagy inducers, resveratrol has attracted attention, recently [21–24]. In our previous study, we confirmed that resveratrol induced autophagy in OSCC cell lines [25]. So, we employed resveratrol as an autophagy inducer and performed AO (acridine orange) staining and western blotting. Our findings demonstrated that resveratrol increased AO-positive autophagic vacuoles, and a typical autophagy inhibitor, 3MA, decreased the number of AO-positive cells. CD also remarkably diminished autophagic vacuoles compared to the resveratrol-treated group (Figure 4(a)). As shown in Figures 4(b) and 4(c), resveratrol upregulated autophagy-related proteins (ATG5, p62, and LC3B) inhibited by 3MA. CD also inhibited resveratrol-induced autophagy, with 3MA and CD cotreatment more effective against resveratrol-induced autophagy compared to single treatment. The aforementioned results suggested that CD acts as an autophagy inhibitor, while 3MA and CD dual treatment effectively suppressed resveratrol-induced autophagy.

**Figure 5:**
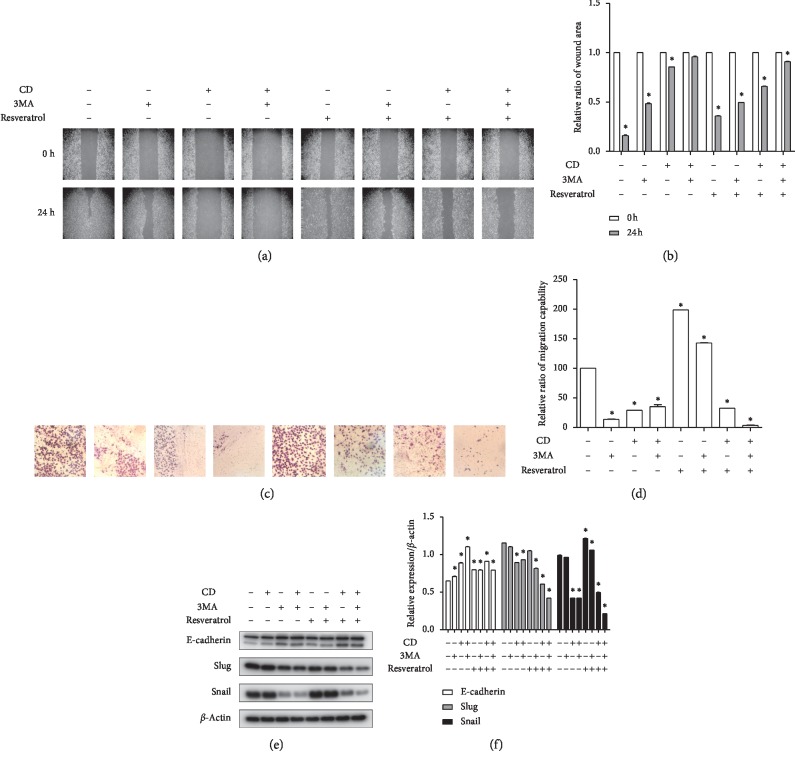
CD and 3MA dual treatment suppressed autophagy-affected cell migration and EMT in Ca9-22 cells. (a) Cells were pretreated with 3MA (1 mM) for 2 h and then 25 *μ*M resveratrol and 50 *μ*M CD for 24 h. Wound area was observed under an inverted microscope by 200x magnification before or after treatment. (b) Relative ratio of healing wound area compared to before treatment. (c) Cells were treated with indicated concentrations and times, and migration capability was detected by transwell assay without Matrigel. (d) Relative ratio of migration capability compared to control. (e) The expression of EMT markers determined by western blot. (f) Relative protein expression levels compared to *β*-actin. Error bars represent mean ± standard deviation (SD). ^*∗*^*p* < 0.05 versus control, respectively.

We hypothesize that CD and 3MA inhibited autophagy-affected migration and EMT in Ca9-22 cells in accordance with previous studies. To validate the hypothesis, wound healing assay and transwell assay (migration assay) were performed. The results indicated that the wound area of the resveratrol-treated group was almost filled with migrated cells. However, the wound area of the 3MA-pretreated and CD-treated groups was not reduced compared to control and resveratrol-administered groups (Figures 5(a) and 5(b)). Similarly, cell migration was induced by resveratrol treatment, and this induction was inhibited by 3MA and CD. 3MA and CD cotreatment significantly inhibited cell migration as shown in Figures 5(c) and 5(d). Next, to further elucidate the effect of CD-inhibited autophagy in the OSCC EMT, we analyzed the EMT-related proteins using a western blot assay. As shown in Figures 5(e) and 5(f), E-cadherin, an epithelial marker, was downregulated by resveratrol. Decreased E-cadherin expression was not regulated by 3MA, but E-cadherin expression increased with application of CD. Meanwhile, the expression levels of slug and snail, with mesenchymal properties, increased with resveratrol. Overexpression of slug and snail was inhibited by 3MA and CD. Additionally, dual treatment of 3MA and CD was more effective with respect to EMT protein expression inhibition than treatment alone. These data suggest that the application of CD effectively suppressed resveratrol-induced OSCC EMT and CD could be a novel autophagy inhibitor.

## 4. Discussion

Over the past several decades, numerous dietary plant-extracted medicines have been used for human cancer. Although various evidence has indicated the advantages of many natural compounds, the OSCC five-year survival rate has been lower (approximately less than 50%) owing to many other reasons [26–28]. Among natural compounds, the *Cudrania tricuspidata* root bark isolated components, having a xanthone skeleton, have exhibited various biological activities [19, 29, 30]. Cudraxanthone H, with a prenylated xanthone, impacts OSCC growth inhibition and apoptosis [16]. Also, gerontoxanthone A, cudraflavone B, and gericudranin E obtained from the root bark of *Cudrania tricuspidata* have hepatoprotective effects [31]. Otherwise, plenty of xanthone series compounds have been used for various studies [26, 32–35]. Yet, there is no report on CD, which possesses a xanthone skeleton with a hydrogen-combined hydroxyl group and mutually *ortho*-located hydroxyl groups, elements of root bark of *Cudrania tricuspidata* in human OSCC [15]. In this study, for the first time, we focused on antimetastatic effects and chemotherapeutic potential of CD against OSCC.

First, we explored the cytotoxic effects of CD on four types of OSCC. Applications of 0 to 100 *μ*M concentrations of CD inhibited cell growth in a dose- and time-dependent manner. Among them, Ca9-22 and SCC25 cells were more sensitive to CD than other cell lines. Recurrence and mortality rates of cancer mostly were regulated by metastasis and invasion of tumor cells, especially OSCC that chiefly metastasizes to the lymph nodes of cervical regions, fatal during prognosis [36–38]. Additional experiments such as wound healing assay, transwell assay, and western blot to detect expression levels of EMT-related protein indicated that CD remarkably suppressed cell migration, invasion, and EMT in Ca9-22 and SCC25 cells. To investigate the EMT inhibition mechanisms of CD, we concentrated on autophagy-regulated Ca9-22 cell EMT.

More research studies have reported that autophagy has a close correlation with cancer cell migration and invasion capability. A previous study suggested that compressive stress upregulates autophagy, and this status leads to enhanced migration of HeLa cells [39]. Further, Gao et al. reported that glycochenodeoxycholate supports HCC cell invasion and migration through autophagy activation [40]. Moreover, inhibition of autophagy with chloroquine attenuates metastatic ability in human non-small-cell lung adenocarcinoma A549 cells [5]. For these reasons, we formulated a hypothesis that CD-inhibited cell EMT ability is related to autophagy in OSCC cells. As an autophagy inducer, resveratrol is used on the basis of a number of earlier studies [21, 23, 41–43]. In our previous study, we evaluated the noncytotoxic concentration of resveratrol [25], where we induced autophagy by 25 *μ*M of resveratrol.

As expected, resveratrol induced AO-positive autophagic vesicles and expressions of autophagy markers (ATG5, p62, and LC3B). 3MA, known as a typical autophagy inhibitor, suppressed resveratrol-induced autophagic activity. CD also inhibited resveratrol-induced autophagic actions. In order to elucidate the mechanism between resveratrol-induced autophagy and OSCC migration as well as invasion capability, we performed wound healing assay, transwell assay without Matrigel, and a western blot assay (EMT-related factors) under the same conditions as in Figure 4. The results demonstrated that autophagy induction enhanced cell migration ability and EMT factor expressions in Ca9-22 cells. Inhibition of autophagy by 3MA decreased Ca9-22 cell motility and EMT-related protein expression compared to the resveratrol-treated group. Suppression of EMT by CD was more effective than that by 3MA. Additionally, 3MA and CD cotreatment dramatically inhibited EMT compared with a single treatment. These results suggested that CD could be an EMT suppressor through autophagy inhibition.

## 5. Conclusion

The present study demonstrated that the molecular mechanism underlying the chemotherapeutic properties is that CD could inhibit metastatic related phenotype in human OSCC cells by inhibiting autophagy. We are suggesting that CD has sufficient potential for development as a new anticancer drug.

## Data Availability

The data used to support the findings of this study are available from the corresponding author upon request.
